# Quantifying Similarities Between MediaPipe and a Known Standard to Address Issues in Tracking 2D Upper Limb Trajectories: Proof of Concept Study

**DOI:** 10.2196/56682

**Published:** 2024-12-17

**Authors:** Vaidehi Wagh, Matthew W Scott, Sarah N Kraeutner

**Affiliations:** 1 Neuroplasticity, Imagery, and Motor Behaviour Lab, Department of Psychology, University of British Columbia, Kelowna, BC, Canada; 2 School of Kinesiology, University of British Columbia, Vancouver, BC, Canada

**Keywords:** markerless pose estimation, procrustes analysis, artificial intelligence, motion, movement tracking, touchscreen, markerless tracking, upper limb, motor

## Abstract

**Background:**

Markerless motion tracking methods have promise for use in a range of domains, including clinical settings where traditional marker-based systems for human pose estimation are not feasible. Artificial intelligence (AI)–based systems can offer a markerless, lightweight approach to motion capture. However, the accuracy of such systems, such as MediaPipe, for tracking fine upper limb movements involving the hand has not been explored.

**Objective:**

The aim of this study is to evaluate the 2D accuracy of MediaPipe against a known standard.

**Methods:**

Participants (N=10) performed a touchscreen-based shape-tracing task requiring them to trace the trajectory of a moving cursor using their index finger. Cursor trajectories created a reoccurring or random shape at 5 different speeds (500-2500 ms, in increments of 500 ms). Movement trajectories on each trial were simultaneously captured by the touchscreen and a separate video camera. Movement coordinates for each trial were extracted from the touchscreen and compared to those predicted by MediaPipe. Specifically, following resampling, normalization, and Procrustes transformations, root-mean-squared error (RMSE; primary outcome measure) was calculated between predicted coordinates and those generated by the touchscreen computer.

**Results:**

Although there was some size distortion in the frame-by-frame estimates predicted by MediaPipe, shapes were similar between the 2 methods and transformations improved the general overlap and similarity of the shapes. The resultant mean RMSE between predicted coordinates and those generated by the touchscreen was 0.28 (SD 0.06) normalized px. Equivalence testing revealed that accuracy differed between MediaPipe and the touchscreen, but that the true difference was between 0 and 0.30 normalized px (*t*_114_=−3.02; *P*=.002). Additional analyses revealed no differences in resultant RMSE between methods when comparing across lower frame rates (30 and 60 frames per second [FPS]), although there was greater RMSE for 120 FPS than for 60 FPS (*t*_35.43_=−2.51; *P*=.03).

**Conclusions:**

Overall, we quantified similarities between one AI-based approach to motion capture and a known standard for tracking fine upper limb movements, informing applications of such systems in domains such as clinical and research settings. Future work should address accuracy in 3 dimensions to further validate the use of AI-based systems, including MediaPipe, in such domains.

## Introduction

Within clinical and rehabilitation settings, the evaluation and monitoring of an individual’s motor function and impairment is important when assessing the effectiveness of an intervention [1]. Standardized clinical assessments, while highly important, often involve a degree of subjectivity (eg, [2]). For example, observing and rating movement quality based on a set criterion or scale may be susceptible to bias and inaccuracies [3]. Alternatively, motion capture systems can be used to provide additional and more objective measurements of movement [4,5]; however, challenges may arise due to the cost and maintenance of equipment [6]. Accordingly, a cost and time-effective way in which movement can be evaluated with high accuracy is desirable in clinical settings.

Marker-based motion tracking involves optical, mechanical, magnetic, or inertial systems that need complex computational processing and resources [7]. Further, the use of such marker-based systems may be limited when assessing individuals who experience sensory difficulties (eg, tactile sensitivity) and may require customized approaches. Advances in computer vision technology have enabled “marker-less” systems, eliminating the need for physical tracking equipment [8]. While well-established, research-grade markerless systems exist, balancing resources, cost, and output are important to consider when using motion-tracking software. The development of artificial intelligence (AI)–based systems for motion tracking, such as OpenPose [9], DeepLabCut [10], and MediaPipe [11] are “lightweight” approaches that allow the tailoring of computational difficulty and accuracy according to their application.

Such systems have been used for human pose estimation [12], object detection [13], and image segmentation [14], with applications to a range of domains (see [15-18] for examples). In clinical research settings, emerging work has focused on applications of AI-based motion tracking in rehabilitation [19,20], physiotherapy [21], action and posture recognition [22,23], human gait analysis [24], as well as diagnostics in telehealth [25]. Outcome measures of research studies in these domains include trajectory analysis, range of motion analysis, task-based assessment, and accuracy of classification models. For instance, MediaPipe has been applied to gross upper limb movements and tremor identification (eg, [26-29]). Yet, this specific tool has not been optimized and validated for behavioral investigations of fine upper-limb movements. Interestingly, previous research has validated AI-based motion capture for gross motor tracking, such as running [30] and stationary cycling [31], as well as in hip, knee, shoulder, and elbow joint movements [32]. Fine upper-limb movements differ from these applications due to a large diversity in parameters relating to dexterity, speed, occlusions, overlaps that occur during movement and lower contrast patterns between individual features as compared to gross upper limb movements [33]. Given the number of degrees of freedom, and complexity in assessing such fine movements, it is critical to determine and quantify the accuracy of AI-based systems, in comparison with known standards. Validation of these systems for the purpose of tracking fine upper-limb movements would provide a key step toward cost-effective motion tracking in clinical settings, particularly for populations with upper-limb movement impairments (eg, individuals after stroke [34], individuals with Parkinson disease [35], cerebral palsy [36], or developmental coordination disorder [37]).

Accordingly, this work aimed to evaluate whether the model solutions given by one AI-based approach to motion tracking, MediaPipe, can be used for accurate tracking of 2D, fine upper limb movements. To address this objective, we evaluated the 2D accuracy of MediaPipe against a known standard, using a touchscreen-based shape-tracing task. Specifically, this task was used to generate 2D trajectories of hand or arm movements on a touchscreen computer. Videos of the hand or arm movements were processed through MediaPipe to obtain predicted coordinates of the movement. Additional postprocessing steps (resampling and normalization) were applied to standardize predicted data by moving it into a common reference space. To assess the accuracy of these predictions, the processed 2D coordinate data were compared to our known standard; coordinates obtained from the touchscreen computer. Following Procrustes transformations to facilitate comparison, root-mean-squared error (RMSE; our primary outcome measure) was obtained between predicted coordinates and those generated by the touchscreen computer. We hypothesized that the predicted coordinates would be equivalent to those generated by the touchscreen computer.

## Methods

### Participants

Data were collected from 10 young healthy adults (aged mean 19.5, SD 1.3 years; 9 females, 1 male, 9 right-handed, and 1 ambidextrous) who participated in the study. All participants had normal or corrected-to-normal vision and were free of neurological disorders or any physical impairment that would impact upper-limb movement. Participants were recruited from the Institution’s Undergraduate Psychology Research Participation Pool.

### Ethical Considerations

Informed consent was obtained from all participants. Ethics approval was obtained from the University of British Columbia Okanagan’s research ethics board (#H21-02626). All study data were deidentified. Participants were provided with course credit for their research participation, in accordance with the Institution’s Undergraduate Psychology Research Participation Pool system and procedures.

### Behavioral Task

**Figure 1.**
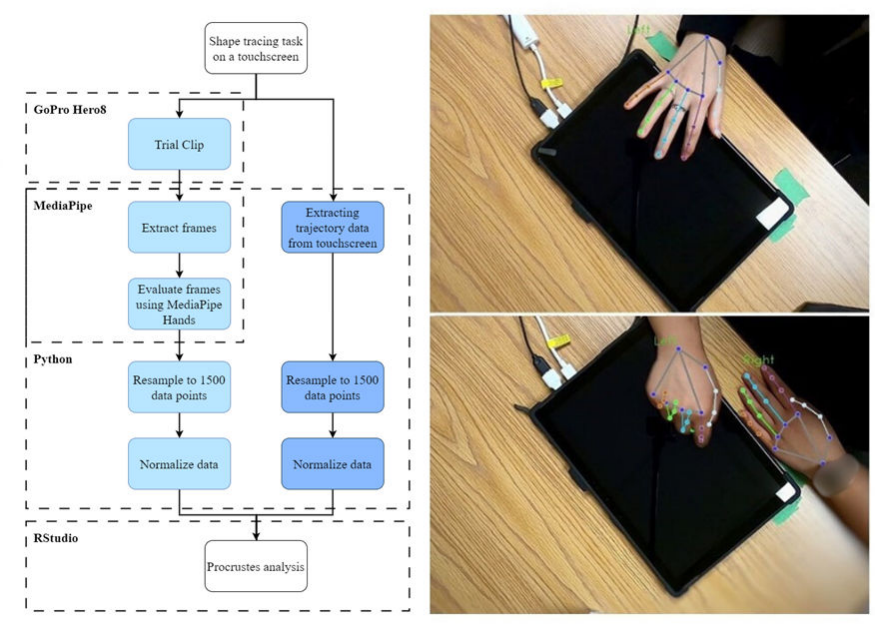
Left: overview of the analysis pipeline applied to data extracted from the touchscreen-based shape-tracing task performed by young healthy adults during a single session, and predicted by MediaPipe from videos obtained simultaneously with a camera (GoPro Hero8). Following video preprocessing (cropped to the length of 1 trial), frames were extracted and evaluated with MediaPipe. Postprocessing (resampling and normalization) was applied to both MediaPipe and touchscreen-based shape-tracing task data, with the two datasets compared using Procrustes transformations and analysis. Right: still-frames from trial clips of the shape-tracing task with MediaPipe Hands landmarks overlaid to illustrate instances where the index finger was visible (top) and partially occluded (bottom).

All participants engaged in a 2D shape-based tracing task performed on a touchscreen computer using custom software developed in the Python programming language (Python Software Foundation) [38]. This task is described in prior work [39]. Briefly, each trial began with the participant tapping a “go” button located on the lower corner of the screen to trigger the movement of a white cursor originating from the starting point. Trajectories consisted of 5 segments (curved paths between 4 additional points and ending at the starting point). All trajectories were animated clockwise from point to point, with no visual feedback (“trace”) left on the screen for participants to track. The duration of each animation ranged between 500 milliseconds and 2500 milliseconds, in increments of 500 milliseconds (ie, 5 different speeds; [39]), and was randomized across trials. Immediately following the animation, participants were instructed to reproduce the trajectory on the screen beginning and ending from the starting point and were instructed to match the speed at which it was animated. All participants performed the task in a seated position directly in front of a horizontally oriented 12.3-inch touchscreen (Microsoft Surface Pro 7) placed on a desk. Participants performed the task with the index finger of their dominant hand, with their nondominant hand resting comfortably in their lap. All participants performed these tasks in blocks, comprising 10 trials each. The x-y coordinates of the participant’s produced trajectory for each trial were recorded by the computer and stored for offline analysis. To obtain videos of the shape-tracing task, a camera (GoPro Hero8; GoPro Inc) was mounted directly above the touchscreen computer to simultaneously record the performance of each trial. The camera position remained fixed throughout the session and was constant across participants, and captured movements in an “overhead” view (Figure 1). One video was recorded per block (10 trials) and stored for offline analysis.

### Data Analysis

#### Behavioral Task

Preprocessing of the touchscreen-based shape-tracing task data occurred as described in Ingram [39]. Preprocessing was performed to standardize participant’s produced trajectories by optimally transforming response trajectories onto the original stimulus (animation) trajectories, to account for variability in timing (via dynamic time warping [40]; to allow for natural variation in movement speed dynamic time warping) and natural variations in movement (via Procrustes transformation [41]; to account for “shape accuracy” independent of translation, rotation, and scale).

#### Video Analysis

Videos were captured by a GoPro Hero8. Each video was cropped to the length of 1 individual trial (ie, 1 complete trial per video), resulting in clips that ranged between 2 and 6 seconds (ie, as participants performed the task at different speeds). A total of 12 blocks of trials were chosen for final analysis, including 1 to 2 blocks for each participant. In total, 5 trials were excluded due to participant compliance, leaving 115 trials in our final analyses. OpenCV, an open-source computer vision and machine learning library [42] was used to extract frames from each 2‐6 second clip in the form of “.jpg” images. A mean of 174 frames was obtained per video depending on the duration of the participant’s tracing movement in each 2‐6 second clip. These frames were evaluated using the MediaPipe Hands solution for Python.

#### MediaPipe Hands

MediaPipe Hands is a customizable, high-fidelity hand and finger tracking solution that uses machine learning to detect 21 landmarks of a hand with right and left-handedness detection [43]. Specifically, the landmarks that are detected are the tip of each finger (4); metacarpophalangeal, proximal interphalangeal, and distal interphalangeal joints of the 4 fingers (12); the tip of the thumb (1); carpometacarpal, metacarpal, and interphalangeal joints of the thumb (4); and the wrist (1) [44]. MediaPipe Hands uses a single shot palm detection model to detect hands in the input image, followed by a hand landmark detector model over the bounding box of the detected hand for precise key point location of 21 fingertip and knuckle coordinates in virtual coordinates and real-world coordinates. The model appears robust to self-occlusions, partially visible hands, and multiple instances of hands in a given image (eg, see ). For this experiment, the MediaPipe Hands solution was deployed in a Google Colab environment [45] to evaluate the input frames. The frames for each trial clip were iterated through and evaluated serially by the MediaPipe Hands model, and x-y coordinates for dominant hand index fingertips were extracted frame-by-frame. This coordinate data were stored pertaining to each trial trajectory. The coordinates were captured between 0‐1920 units of x coordinates and 1080 units of y coordinates for an image resolution of 1920×1080. The x-y coordinates of the index fingertip output for each trial were then postprocessed.

#### Postprocessing

Our overall pipeline is shown in Figure 1. Output x-y coordinates for all trials were resampled to 1500 points using univariate spline interpolation with k=3 (cubic spline interpolation, via the “interpolated univariate spline” function from the “scipy.interpolate” module in Python; [46]), to interpolate between predicted points. These coordinates were obtained within the coordinate system bounded by the height and width of the input image, between axis limits of 1920×1080. Resampled data were then normalized by minmax scaling in Python using the sklearn.preprocessing module [47] to obtain the final predicted coordinate data between 0 and 1 for each trial. To facilitate an accurate comparison of MediaPipe predictions with trajectories extracted from the touchscreen, coordinates generated from the touchscreen-based shape-tracing task were postprocessed as described. Specifically, trajectories extracted from the touchscreen-based shape-tracing task were also resampled to 1500 points, via the scipy.interpolate module, and normalized using the sklearn.preprocessing library given differences in reference frames and coordinate systems between MediaPipe and the touchscreen.

Once data from both MediaPipe predictions and the touchscreen-based shape-tracing task were extracted, resampled (1500 points) and normalized (range of 0 to 1), the 2 datasets were compared to quantitatively assess the accuracy of the predictions using Procrustes transformations and analysis in R programming environment (version 4.2.1; R Core Team) [48] via the vegan package [49]. Specifically, geometric transformations are applied to the MediaPipe predicted trajectory to rotate, scale, and translate the dataset to best match it to the touchscreen trajectory. The best match is determined by the lowest RMSE between the corresponding points of the 2 datasets:


$$\begin{array}{l} RMSE=\sqrt{\sum_{i=1}^{n}\frac{{\left(M_{original}-\;M_{transformed}\right)}^{2\;}}{n}\;}\;=\\ \;\sqrt{\sum_{i=1}^{n}\frac{\left({\left(X_{original}-\;X_{transformed}\right)}^{2\;}+{\left(Y_{original}-\;Y_{transformed}\right)}^{2\;}\right)}{n}} \end{array}$$

Here, M_original_ is a set of x and y coordinates extracted from the touchscreen. M_transformed_ is the corresponding set of x and y coordinates predicted by MediaPipe, following Procrustes transformations. *n* is the total number of coordinates data points extracted (here, 1500).

**Figure 2.**
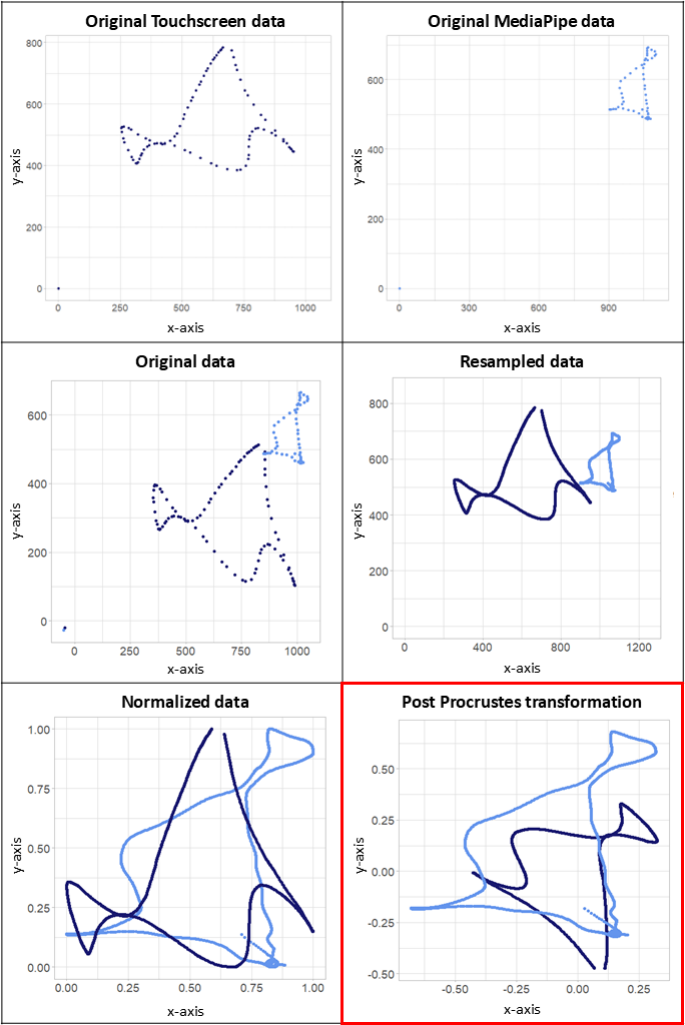
Data from an exemplar shape-tracing trial of the touchscreen-based shape-tracing task are plotted at each step in the methodology. Top row: the original trace extracted from the touchscreen (dark blue) and predicted by MediaPipe (light blue) are shown. Middle row: the raw traces are then merged into the same plot (left). Resampling is then applied to both datasets (right). Bottom row: datasets are then normalized (left), with Procrustes transformations applied for comparison (right).

Figure 2 shows an exemplar trace extracted from the touchscreen and generated from MediaPipe with postprocessing steps applied for illustrative purposes. Applying this Procrustes transformation and analysis, we obtained RMSE for each pair of coordinates predicted by MediaPipe and the data from our touchscreen computer (our known standard) for each trial.

### Statistical Analysis

#### Accuracy of MediaPipe

Equivalence tests were performed on the resultant RMSE calculated for coordinates generated by MediaPipe versus the touchscreen computer, to determine whether the predicted coordinates were equivalent to touchscreen-generated coordinates. Specifically, an equivalence test was performed (via two one-sided tests; α<.05) on RMSE using the TOSTER package in R Programming Environment [50]. These tested the null hypotheses that the true mean is equal to 0 (null hypothesis significance testing), and the true mean is more extreme than 0 and 0.30 (two 1-sided tests). A threshold of 0.3, representing the minimum effect (magnitude of difference) of interest, was used based on Wade et al [51] whereby an AI-based pose estimation approach used to track a lower limb movement was compared to a manual tracking system. The mean absolute resultant effects for markers on the ankle, calcaneus, and fifth metatarsal head was 0.35. As a more conservative estimate given that our task involved an upper-limb movement, we decreased this threshold from 0.35 to 0.3.

#### Impact of Frame Rates on Resultant RMSE

To explore the impact of the frame rate parameter on the accuracy of landmark prediction, we assessed 2 blocks of trials (ie, 20 video clips) for 3 different frame rates: 30, 60, and 120 frames per second (FPS). Pairwise *t* tests were conducted to determine if RMSE differed across frame rates. Specifically, 2 separate *t* tests were conducted to compare RMSE between videos of 30 and 60 FPS, and between videos of 60 and 120 FPS, with Bonferroni corrections applied for multiple comparisons.

## Results

### Accuracy of MediaPipe

Visual inspection of our results on a frame-by-frame basis suggested that using MediaPipe to capture trajectories was overall similar to trajectories obtained from the touchscreen in short-duration clips, yet that postprocessing resulted in greater overlap and improved the comparison. The resultant mean RMSE between trajectories predicted by MediaPipe and those captured by the touchscreen was 0.28 (SD 0.06) px. Our statistical tests conducted (testing the null hypotheses that true mean is equal to 0, and the true mean is more extreme than 0 and 0.30) indicated that RMSE was not equal to 0 (*t*_114_=47.552; *P*<.001); yet, that at the selected error rate the true mean was found to be between 0 and 0.3 (*t*_114_=−3.02; *P*=.002; 90% CI 0.27-0.29; Hedges *g*=4.40; 90% CI 3.96-5.0).

### Impact of Frame Rate on Resultant RMSE

The mean RMSE across frame rates are reported in Table 1. From our analysis conducted to determine if resultant RMSE (ie, between MediaPipe and the touchscreen) differed across frame rate, no differences were observed between video clips of 30 and 60 FPS (*t*_35.05_=0.707; *P*=.97; 95% CI −0.02 to 0.04); yet, video clips of 120 FPS resulted in greater RMSE than video clips of 60 FPS (*t*_35.43_=−2.51; *P*=.03; 95% CI −0.07 to −0.01).

**Table 1.** Mean RMSE^a^ calculated between trajectories predicted by MediaPipe and extracted from the touchscreen computer across 30, 60, and 120 frames per second. RMSE was assessed between 2 datasets for each individual trial following postprocessing, with Procrustes transformations applied.

Frame rate, n	Number of trials, n	Mean RMSE (SD)
30	20	0.29 (0.042)
60	20	0.28 (0.056)
120	20	0.32 (0.043)

a RMSE: root-mean-squared error.

## Discussion

As a proof of concept, we evaluated the extent to which trajectories predicted by one AI-based approach to motion tracking, MediaPipe, deviated from those generated by a touchscreen computer (a “gold standard”). In partial support of our hypotheses, while a statistical difference between these trajectories was found, this difference was limited. Specifically, the mean RMSE was found to be 0.28 (SD 0.06) px with the true magnitude of difference to be less than 0.3 via equivalence testing (90% CI 0.27-0.29). These results support the use of MediaPipe in domains where positional data of the hand or arm are required to measure complex upper-limb movements (for example, see [18,20,25-27]). In this study, it appeared possible to extract coordinates when key features of hands are occluded, such as when the palm is occluded by the fingers while making a fist. Extracting these coordinates also appeared possible in the presence of a second hand (ie, the nondominant hand) and from low-resolution or blurred images. However, it is important to consider the trade-off between the accuracy and computational complexity of motion-tracking systems to conclusively determine the benefit of AI-based approaches to motion-tracking applied to research and clinical domains such as physiotherapy, rehabilitation, and telerehabilitation. Eliminating the sole reliance on physical tracking equipment enables motion tracking in a wide variety of clinical domains where it may not always be feasible or possible to use such systems [8]. Validating AI-based markerless systems is a key step toward bridging the gap between the theory and practice of remote clinical assessment [51].

Our results represent a necessary step to address this gap, by quantifying the accuracy of 1 markerless system used for fine upper-limb tracking in 2D space, informing on future applications of AI-based approaches to motion capture in these domains. Our pipeline evaluated videos of 2‐6 seconds in duration with each video representing 1 trial, with a mean of 174 frames evaluated for each video. It is likely that the reported error can be attributed to any difference between the fingertip landmark predicted in this study, to where the finger is sensed on the touchscreen. While deviations (likely distal) may result in different positional estimates, in this instance a comparable trajectory assessment of within-video change would be maintained. Therefore, while between-subject comparisons of error should be made with caution, our findings suggest that MediaPipe may be suitable for assessing relative within-person change in upper limb movements over the course of an intervention or training regime. Any distortions during video capture, approximated geometric transformations, or low-resolution data capture may also contribute to the reported error.

Palucci Vieira et al [52] compared an AI-based motion tracking system (OpenPose) to a manual tracking system. No differences were observed between approaches when tracking the position of the hip or knee. Yet, for the position of the ankle, heel, and fifth metatarsal head, which can be considered finer lower limb movements, small effects (absolute mean of 0.35) between the approaches were observed. AI-based systems, including MediaPipe, may thus perform better for gross movements, including that of the torso or postural sway, head, or hip movements rather than fine upper limb movements as in our study. While we used a threshold of 0.3 (ie, such that we aimed to statistically reject effects larger than 0.3) it is important to practically consider different maximum allowable effects to assess the sensitivity of such systems when tracking the position of different effectors and across various use cases. For instance, Biswas et al [53] used physical sensors (accelerometers and gyroscopes) to detect 3 different upper limb movements typically performed during activities of daily living (eg, making tea). While accuracy ranged from 40% to 88% across both types of sensors, authors suggested the average resultant accuracy (66%, gyroscope and 70%, accelerometer) was determined to be effective given that such movements were performed in naturalistic settings. With respect to the approach taken in this study, MediaPipe has also been validated against accelerometers for measuring upper limb tremors in individuals with Parkinson [27]. Low resultant error (mean absolute error 0.229, SD 0.174 Hz), and a high correlation in amplitude measurement were shown when comparing MediaPipe to the physical sensors [27]. Indeed, alternative well-established markerless systems are available (eg, Vicon and Kinect), representing high-quality solutions that can eliminate the need for physical tracking systems. Given that this investigation compared one AI-based motion capture tool to a touchscreen computer (representing our known standard), comparing AI-based systems to established markerless tools and considering the sensitivity of different AI-based approaches is an important avenue of future research.

Our secondary analysis probed the impact of the frame rate parameter to increase the accuracy of the pipeline. In our pipeline, a low frame rate of 30 FPS resulted in similar accuracy, but counterintuitively, a higher frame rate of 120 FPS resulted in increased tracking error. This result may be due to the increased number of resampling points. Past work has demonstrated MediaPipe-based tracking with 11 and 20 FPS [54] and with 30 FPS for full body posture detection (with additional processing applied [ie, to correct for incorrect depth estimation, jitter, and lag]; [22]) and motor skill assessment of the hand (with additional depth correction) [26]. While future research is needed to determine the impact of resampling on videos of higher FPS, our findings support the use of consumer-grade devices where 60 FPS is typical. Our findings also suggest that postprocessing steps should be included in an optimal processing pipeline for investigations of fine upper-limb movement using MediaPipe. Here, postprocessing methods including resampling and normalization improved transformation output. In particular, this method adjusted outputs to a common coordinate system to enable comparison across different systems (as in this study, between MediaPipe and the touchscreen computer), or to provide a method of standardization across participants. Normalization can further help eliminate the need for shifting coordinate systems before comparison. Customizing the postprocessing pipelines according to a particular task may thus be important to the application of MediaPipe in different domains.

It is important to consider that our investigation comprised a sample of healthy young adults and was restricted to 2D coordinates. Showing the 2D accuracy of MediaPipe represents an important first step toward its validation. While our findings are encouraging for the use of AI-based alternatives, such as MediaPipe, for assessing movement kinematics in healthy and clinical populations (including those with fine upper limb motor impairment, eg, individuals after stroke, with cerebral palsy, or with developmental coordination disorder) where assessing change in kinematics over the course of an intervention can be used to track improvements in motor function, future work is needed to assess its accuracy and feasibility for tracking 3D fine upper-limb movements. Furthermore, AI-based motion tracking tools may provide a fruitful avenue for the assessment of home-based, upper-limb focused, therapies, which have been shown to be effective (eg, [55-58]). For instance, individuals engaging in physical therapy may record themselves performing a task via mobile phone and send these to their therapist for documentation and assessment. As we tested one AI-based approach to motion tracking in a laboratory setting, testing these tools in more ecological settings is an important next step that may enable motion tracking in variable environments (ie, differing backgrounds, obstructions, and light illuminations).

While MediaPipe was trained on a synthetically developed dataset consisting of hand models layered with 5 hand textures and skin tones and natural image datasets to mitigate racial bias [43], past work has shown that skin tones may affect MediaPipe reliability when tracking a surgical technique [54]. Given that racial bias has been noted in deep learning models [59], future work should consider the impact of factors such as race and skin tone of the participants on the accuracy of predictions generated by AI-based motion capture systems applied to fine upper limb movements. Further, given our main aim related to testing the accuracy of MediaPipe (ie, rather than assessing behavioral change of individual participants), we considered each video (ie, movement) as an independent observation in our analysis. As our videos were not used to train the model and each video was treated as a new exposure, extracted coordinates were not an extrapolation predicated on previous trials, and the speed at which each movement was completed varied to reduce the likeness of videos. The robustness of the models to such variances is a crucial feature of motion tracking systems to ensure applicability in diverse settings. However, future work using MediaPipe to assess behavioral change over the course of an intervention should consider the use of mixed models such that random effects can be entered to account for multiple videos extracted from the same participant (ie, trials across a test block). Exploring this approach with our data, we simulated a dataset with a mean RMSE of 0.3 and conducted a linear mixed effects model on RMSE by type (actual and simulated). While this analysis tests for a difference (rather than equivalence), no effects were observed, thus supporting findings from our original analysis approach (see Multimedia Appendix 1).

In this study, we assessed the 2D accuracy of one AI-based markerless approach to motion tracking, MediaPipe, against a known standard. Our findings show an equivalence (within a range of 0-0.30 normalized px) between trajectories predicted by MediaPipe and those extracted from a touchscreen-based shape-tracing task. Our secondary analysis assessing the impact of frame rate supports the use of widely available devices where a frame rate of 60 FPS is typical. Future work should address low-cost motion capture using 2D image data for 3D pose estimation to further validate the use of “lightweight” alternatives to motion tracking in clinical populations and different settings (eg, clinical and diagnostic). While this work represents a necessary step toward validating the use of AI-based motion capture systems in investigations of fine upper-limb movement, future work is required to assess accuracy for tracking 3D fine upper-limb movements across a larger sample.

## Supplementary material

10.2196/56682 Multimedia Appendix 1 Exploratory analysis.

## Abbreviations

AI: artificial intelligence
FPS: frames per second
RMSE: root mean squared error

## References

[1] Nascimento L do, Bonfati LV, Freitas MLB, Mendes Junior JJA, Siqueira HV, Stevan SL (2020). Sensors and systems for physical rehabilitation and health monitoring—a review. Sensors (Basel).

[2] Hadwin KJ, Wood G, Payne S, Mackintosh C, Parr JVV (2023). Strengths and weaknesses of the MABC-2 as a diagnostic tool for developmental coordination disorder: an online survey of occupational therapists and physiotherapists. PLoS ONE.

[3] Lincoln NB, Crow JL, Jackson JM, Waters GR, Adams SA, Hodgson P (1991). The unreliability of sensory assessments. Clin Rehabil.

[4] Khaksar S, Pan H, Borazjani B (2021). Application of inertial measurement units and machine learning classification in cerebral palsy: randomized controlled trial. JMIR Rehabil Assist Technol.

[5] Ota H, Mukaino M, Inoue Y (2023). Movement component analysis of reaching strategies in individuals with stroke: preliminary study. JMIR Rehabil Assist Technol.

[6] Selvaraj S, Sundaravaradhan S (2020). Challenges and opportunities in IoT healthcare systems: a systematic review. SN Appl Sci.

[7] Zhou H, Hu H (2008). Human motion tracking for rehabilitation—a survey. Biomed Signal Process Control.

[8] Lam WWT, Tang YM, Fong KNK (2023). A systematic review of the applications of markerless motion capture (MMC) technology for clinical measurement in rehabilitation. J Neuroeng Rehabil.

[9] Cao Z, Simon T, Wei SE, Sheikh Y Realtime multi-person 2d pose estimation using part affinity fields.

[10] Mathis A, Mamidanna P, Cury KM (2018). DeepLabCut: markerless pose estimation of user-defined body parts with deep learning. Nat Neurosci.

[11] Lugaresi C, Tang J, Nash H (2019). Mediapipe: a framework for building perception pipelines. Google Research.

[12] Singh AK, Kumbhare VA, Arthi K Real-time human pose detection and recognition using mediapipe.

[13] Ahmadyan A, Hou T, Wei J, Zhang L, Ablavatski A, Grundmann M (2020). Instant 3D object tracking with applications in augmented reality. arXiv.

[14] Dijkstra-Soudarissanane S, Gunkel SN, Reinders V Virtual visits: life-size immersive communication.

[15] Halder A, Tayade A (2021). Real-time vernacular sign language recognition using mediapipe and machine learning. J homepage.

[16] Zhang M, Schulze J, Zhang D (2021). Faceatlasar: atlas of facial acupuncture points in augmented reality. arXiv.

[17] Karg P, Kreimeier J, Götzelmann T Build-and-touch: a low-cost, DIY, open-source approach towards touchable virtual reality.

[18] Xiao F, Zhang Z, Liu C, Wang Y (2023). Human motion intention recognition method with visual, audio, and surface electromyography modalities for a mechanical hand in different environments. Biomed Signal Process Control.

[19] Koh WK, Nguyen QH, Yang YO, Xu T, Nguyen BP, Chua MCH (2021). Soft Computing: Biomedical and Related Applications.

[20] Sousa DL, Teixeira S, Fontenele JE (2024). Health care professional-supported co-design of a mime therapy-based serious game for facial rehabilitation. JMIR Serious Games.

[21] Bhatambarekar G, Bhinge SA, Bhale K, Dandgaval A, Ramteke K A study on physiotherapy exercise corrector.

[22] Lakkapragada A, Kline A, Mutlu OC (2022). The classification of abnormal hand movement to aid in autism detection: machine learning study. JMIR Biomed Eng.

[23] Lin Y, Jiao X, Zhao L (2023). Detection of 3D human posture based on improved Mediapipe. JCC.

[24] Gupta A, Shrestha PL, Thapa B, Silwal R, Shrestha R (2023). Knee flexion/extension angle measurement for gait analysis using machine learning solution “MediaPipe Pose” and its comparison with Kinovea ^®^. IOP Conf Ser: Mater Sci Eng.

[25] Latreche A, Kelaiaia R, Chemori A, Kerboua A (2023). Reliability and validity analysis of MediaPipe-based measurement system for some human rehabilitation motions. Measurement (Lond).

[26] Palani P, Panigrahi S, Jammi SA, Thondiyath A Real-time joint angle estimation using mediapipe framework and inertial sensors.

[27] Amprimo G, Ferraris C, Masi G, Pettiti G, Priano L GMH-d: combining google mediapipe and rgb-depth cameras for hand motor skills remote assessment.

[28] Güney G, Jansen TS, Dill S (2022). Video-based hand movement analysis of Parkinson patients before and after medication using high-frame-rate videos and MediaPipe. Sensors (Basel).

[29] Ghanbari S, Ashtyani ZP, Masouleh MT User identification based on hand geometrical biometrics using media-pipe.

[30] Srinivasan HK, Mathunny JJ, Devaraj A, Karthik V Validation of an automated step length measurement method in sprinting athletes using computer vision and pose estimation.

[31] Bini RR, Nascimento VB, Nibali A (2023). Validity of neural networks in determining lower limb kinematics in stationary cycling. Sport Sci Health.

[32] Lafayette TB de G, Kunst VH de L, Melo PV de S (2022). Validation of angle estimation based on body tracking data from RGB-D and RGB cameras for biomechanical assessment. Sensors (Basel).

[33] Ahmad A, Migniot C, Dipanda A (2019). Hand pose estimation and tracking in real and virtual interaction: a review. Image Vis Comput.

[34] Raghavan P (2015). Upper limb motor impairment after stroke. Phys Med Rehabil Clin N Am.

[35] Proud EL, Miller KJ, Bilney B, Balachandran S, McGinley JL, Morris ME (2015). Evaluation of measures of upper limb functioning and disability in people with Parkinson disease: a systematic review. Arch Phys Med Rehabil.

[36] Makki D, Duodu J, Nixon M (2014). Prevalence and pattern of upper limb involvement in cerebral palsy. J Child Orthop.

[37] Grohs MN, Hawe RL, Dukelow SP, Dewey D (2021). Unimanual and bimanual motor performance in children with developmental coordination disorder (DCD) provide evidence for underlying motor control deficits. Sci Rep.

[38] Van Rossum G, Drake FL (2009). Python 3 Reference Manual.

[39] Ingram TGJ, Solomon JP, Westwood DA, Boe SG (2019). Movement related sensory feedback is not necessary for learning to execute a motor skill. Behav Brain Res.

[40] Giorgino T (2009). Computing and visualizing dynamic time warping alignments in R: the dtw package. J Stat Softw.

[41] Kadmon Harpaz N, Flash T, Dinstein I (2014). Scale-invariant movement encoding in the human motor system. Neuron.

[42] Bradski G (2000). The openCV library. Dr Dobb’s J Softw Tools Prof Program.

[43] Zhang F, Bazarevsky V, Vakunov A (2020). Mediapipe hands: on-device real-time hand tracking. arXiv.

[44] Hand landmarks detection guide. MediaPipe Solutions.

[45] Google colaboratory. Welcome to Colab.

[46] Virtanen P, Gommers R, Oliphant TE (2020). SciPy 1.0: fundamental algorithms for scientific computing in Python. Nat Methods.

[47] Pedregosa F, Varoquaux G, Gramfort A, Michel V, Thirion B, Grisel O (2011). Scikit-learn: machine learning in Python. J Mach Learn Res.

[48] R core team: R: A language and environment for statistical computing. R.

[49] Oksanen J, Simpson GL, Blanchet FG, Kindt R, Legendre P, Minchin PR Vegan: community ecology package. R package version 2.6-4. R.

[50] Caldwell AR (2022). Exploring equivalence testing with the updated TOSTER R package. PsyArXiv.

[51] Wade L, Needham L, McGuigan P, Bilzon J (2022). Applications and limitations of current markerless motion capture methods for clinical gait biomechanics. PeerJ.

[52] Palucci Vieira LH, Santiago PRP, Pinto A, Aquino R, Torres R da S, Barbieri FA (2022). Automatic markerless motion detector method against traditional digitisation for 3-dimensional movement kinematic analysis of ball kicking in soccer field context. IJERPH.

[53] Biswas D, Cranny A, Gupta N (2015). Recognizing upper limb movements with wrist worn inertial sensors using k-means clustering classification. Hum Mov Sci.

[54] Müller LR, Petersen J, Yamlahi A (2022). Robust hand tracking for surgical telestration. Int J Comput Assist Radiol Surg.

[55] Chen Y, Abel KT, Janecek JT, Chen Y, Zheng K, Cramer SC (2019). Home-based technologies for stroke rehabilitation: a systematic review. Int J Med Inform.

[56] Welsby E, Berrigan S, Laver K (2019). Effectiveness of occupational therapy intervention for people with Parkinson’s disease: systematic review. Aus Occup Therapy J.

[57] Ortega-Martínez A, Palomo-Carrión R, Varela-Ferro C, Bagur-Calafat MC (2023). Feasibility of a home-based mirror therapy program in children with unilateral spastic cerebral palsy. Healthcare (Basel).

[58] Scott MW, Wood G, Holmes PS, Marshall B, Williams J, Wright DJ (2023). Combined action observation and motor imagery improves learning of activities of daily living in children with developmental coordination disorder. PLoS ONE.

[59] Huang J, Galal G, Etemadi M, Vaidyanathan M (2022). Evaluation and mitigation of racial bias in clinical machine learning models: scoping review. JMIR Med Inform.

[60] TraceLab. GitHub repository.

[61] MediaPipe. GitHub repository.

