# Supplementary material for: Quantifying Similarities Between MediaPipe and a Known Standard to Address Issues in Tracking 2D Upper Limb Trajectories: Proof of Concept Study
Source: JMIR Form Res. 2024 Dec 17;8:e56682. doi: 10.2196/56682 (PMC11683656; doi:10.2196/56682)
Supplement: Multimedia Appendix 1 [file formative-v8-e56682-s001.docx]

To explore potential issues related to non-independence of observations, we randomly generated 115 RMSE samples (equalling the number of videos in our analysis) with a mean of these samples equal to 0.3 (true mean of this sample distribution = 0.2997944). These simulated values were entered into our data set, such that we dummy coded a new independent variable termed ‘Type’ with two levels (Actual extracted, Simulated) reflecting our actual (RMSE extracted values used in our equivalence test) and ‘true’ values (simulated values with a mean of 0.3, representing the true mean tested in our equivalence test). To test if RMSE differed as a function of Type a linear mixed effects model was conducted on RMSE, with Type entered as a fixed effect (reference level = True mean values), and participant entered as a random intercept (i.e., accounting for the potential issue of non-independence). No effect of Type was observed.

**Table S1.** Linear mixed effects model conducted on RMSE by data Type (Simulated values with a mean of 0.3, Actual extracted values).

|  | RMSE | | |
| --- | --- | --- | --- |
| *Predictors* | *Estimates* | *CI* | *p* |
| (Intercept) | 0.30 | 0.28 – 0.32 | <0.001 |
| Type [Actual] | -0.02 | -0.04 – 0.00 | 0.122 |
| Random Effects | | | |
| σ^2^ | 0.01 | | |
| τ_00_ _Participant_ | 0.00 | | |
| ICC | 0.04 | | |
| N _Participant_ | 12 | | |
| Observations | 230 | | |
| Marginal R^2^ / Conditional R^2^ | 0.010 / 0.046 | | |
